# Factors associated with major adverse kidney events in patients who underwent veno-arterial extracorporeal membrane oxygenation

**DOI:** 10.1186/s13613-020-00656-w

**Published:** 2020-04-20

**Authors:** Camille Vinclair, Etienne De Montmollin, Romain Sonneville, Jean Reuter, Jordane Lebut, Radj Cally, Bruno Mourvillier, Mathilde Neuville, Stéphane Ruckly, Jean-François Timsit, Lila Bouadma

**Affiliations:** 1 Medical and Infectious Intensive Care Unit, Bichat Claude Bernard University Hospital, AP-HP, 46 rue Henri Huchard, 75018 Paris, France; 2 UMR 1137-IAME Team 5–DeSCID: Decision SCiences in Infectious Diseases control and care INSERM/Univ Paris Diderot, Sorbonne Paris Cité, 75018 Paris, France; 3 Medical Intensive Care Unit, Robert Debré University Hospital, rue du Géneral Koening, 51000 Reims, France

**Keywords:** Extracorporeal membrane oxygenation, Acute kidney injury, Major adverse kidney events, Chronic kidney disease

## Abstract

**Objective:**

To describe acute kidney injury (AKI) natural history and to identify predictors of major adverse kidney events (MAKE) within 1 year in patients supported by veno-arterial extracorporeal membrane oxygenation (VA-ECMO).

**Design:**

Retrospective observational study.

**Setting:**

Medical French intensive care unit between January 2014 and December 2016.

**Patients:**

Consecutive patients implanted with VA-ECMO ≥ 16 years, VA-ECMO for at least ≥ 48 h, and without end-stage chronic kidney disease (CKD).

**Intervention:**

None.

**Measurements:**

Multivariate logistic regression of factors associated with MAKE at 1 year defined as one of the following criteria within day 360: death and receipt of renal replacement therapy (RRT) or persistent renal dysfunction, i.e., CKD ≥ stage 3 corresponding to an estimated glomerular filtration rate (eGFR) ≤ 60 ml/min/1.73 m^2^ and MAKE at day 30 and day 90 defined as one of the following criteria within day 30 or day 90: death, receipt of renal replacement therapy and serum creatinine ≥ threefold increase.

**Main results:**

158 consecutive patients were included (male sex: 75.9%; median and interquartile range: age: 59 [47–66], Simplified Acute Physiology Score II: 55 [39–66], Sepsis-related Organ Failure Assessment Score: 9 [7–12], time on VA-ECMO: 7.5 [4–12] days). Among them 145 (91.8%) developed an AKI during the intensive care unit (ICU) stay and 85 (53.8%) needed renal replacement therapy (RRT). 59.9% (91/152), 60.5% (89/147) and 85.1% (120/141) evaluable patients had a MAKE-30, MAKE-90 and MAKE-360, respectively. Factors significantly associated with MAKE-360 were eGFR at baseline (odds ratio (OR) 0.98, confidence interval 95% (CI) [0.97;1.00], *p* 0.02), Kidney Disease Improving Global Outcome (KDIGO) stage at cannulation (*p* = 0.03), e.g., stage 3 vs. reference stage 0 OR 10.20 [1.77–58.87], and number of red blood cell (RBC) packs received while under ECMO (OR 1.14, CI 95% [1.01;1.28], *p* = 0.03). At 1 year among the 51 survivors, almost half of the alive patients (*n* = 20/51) had a decline of estimated glomerular filtration (eGFR) > 30% mL/min/1.73 m^2^. Their median eGFR decline was − 26.3% [− 46.6;− 10.7].

**Conclusion:**

Patients undergoing VA-ECMO had a high risk of AKI during the ICU stay. Factors associated with MAKE 360 were mainly eGFR at baseline, KDIGO stage at cannulation and, number of RBC packs received while under ECMO. Among survivors at 1 year, almost half of the alive patients (*n* = 20/51) had a decline eGFR > 30%.

## Introduction

Veno-arterial extracorporeal membrane oxygenation (VA-ECMO) is a technique to provide circulatory assistance for patients suffering from cardiogenic shock [1]. Acute kidney injury (AKI) is a common and major complication directly attributable to any extracorporeal device [2]. Various mechanisms are proposed to explain AKI in patients supported with a VA-ECMO such as underlying disease (cardiogenic shock, cardio-renal syndrome), ischemia/reperfusion, systemic inflammatory response syndrome, fluid overload, hypercoagulable state, and hemolysis.

Acute and long-term outcomes of AKI occurring in intensive care unit (ICU) are well-described. AKI is associated with early and late mortality rising with AKI severity [3–5]. Furthermore, AKI is associated with a long-term risk of chronic kidney disease (CKD) [6, 7].

Long-term risk of CKD in patients treated by VA-ECMO was never analyzed. Because of extended use of this technique, early identification of factors associated with better renal outcome of survivors is urgently needed. This information may help to better define the patients who would benefit the most from ECMO or may inspire prompt prevention initiatives and improve the outcome.

The objectives of this study were to describe the natural history of renal function within 1 year and to identify independent predictors of major kidney events (MAKE) at day 30, day 90 and at 1 year in patients who underwent VA-ECMO.

## Materials and methods

### Study design and setting

We retrospectively analyzed the charts of consecutive patients who received VA-ECMO during their ICU stay in our 20-bed ICU (medical ICU, Bichat-Claude Bernard Hospital, Paris, France, an 800-bed university hospital) between 1st of January 2014 and 31st of December 2016. We included patients older than 16 years, and who were under VA-ECMO for at least ≥ 48 h. Patients who received veno-venous ECMO and patients with pre-hospitalization end-stage renal disease (ESRD) requiring renal replacement therapy (RRT) were excluded. Patients who met inclusion criteria multiple times in different ICU stays within a year were included only once.

### Data collection

Data were mainly collected from electronic medical records or otherwise by asking general practitioner of the patients.

The following data were recorded at ICU admission: age, sex, body max index (BMI), Simplified Acute Physiology Score (SAPSII) [8], the SOFA (Sepsis-related Organ Failure Assessment) score [9], extra-renal comorbidities and basal renal function. We also recorded the reason for ECMO support, the location of cannulation, the site of ECMO cannulation and in case of post-cardiac surgery, indication of surgery and duration of cardiopulmonary bypass (CBP).

Within the ICU stay, we collected ECMO-associated bleeding complications: number of red blood cell (RBC) transfusions, hemopericardium defined as early (within 3 days) or otherwise late or other haemorrhagic shock needing surgery.

We collected also: ICU and hospital length of stay, duration of VA-ECMO support, renal function, need of RRT and survival at each time point (ICU, discharge, day 30, day 90, day 360).

### Definition and classification of AKI

AKI stages were defined according to the current (Kidney Disease Improving Global Outcome) KDIGO guidelines based on elevations in serum creatinine (SCr) from the baseline reference value or requirement of RRT [10]. The reference SCr was obtained in the medical history of the patient. If not possible, we used the lowest Scr within the first week (if no history of chronic renal failure and modification of diet in renal disease (MDRD) > 75 ml/min/1.73 m^2^) or we imputed SCr for a MDRD of 75 ml/min/1.73 m^2^ as recommended in the KDIGO guidelines on AKI.

### Definition of steady-state renal function (baseline and at 1 year)

Baseline renal function at admission and at 1 year were calculated using the simplified MDRD equation based on age, gender, race, and calibration for Scr. Normal renal function was defined as an estimated glomerular filtration rate (eGFR) > 90 mM/min/1.73 m^2^. If eGFR by MDRD was over 90 ml/min/1.73 m^2^, CKD stage was labeled 0/1 as no urinalysis was available to distinguish further between these two stages.

Individuals with decreased levels of eGFR were classified according to the consensual stages of CKD [11].

### Outcome measures

The main goal of the present study was to describe the natural history of renal function within 1 year and to identify the risk factors for major adverse kidney events at day 360 (MAKE-360) defined as one of the following criteria within 1 year: death and receipt of renal replacement therapy (RRT) or persistent renal dysfunction, i.e., CKD ≥ stage 3 corresponding to an eGFR by MDRD ≤ 60 ml/min/1.73 m^2.^

The secondary outcomes were to identify the risk factors for MAKE at day 30 (MAKE-30) and at day 90 (MAKE-90) defined as one of the following criteria within day 30 or day 90: death, receipt of RRT and SCr ≥ threefold increase.

### Ethics

This study was approved by the ethical committee of the French Society of Intensive Care (SRLF).

The ethical committee requested that attending physician received a mail with an information letter to deliver to the patient or to a family member.

The database was declared to the French National Commission of data processing (CNIL).

### Statistical analysis

Quantitative variables are reported as median (interquartile range, IQR) and qualitative variables as number (%). For all the analyses the date of origin (day 0) was the of ECMO cannulation.

We performed a univariate analysis with a logistic regression to determine factors associated with MAKE-360, MAKE-30 and MAKE-90 multivariate analysis included medically relevant items:

eGFR at baseline, KDIGO stage at cannulation, SOFA score without hemodynamic and renal items, activated partial thromboplastin time (aPTT) at cannulation, duration of cardiopulmonary bypass (CBP), number of RBC packs while under ECMO, ECMO duration.

## Results

### Study population

**Fig. 1:**
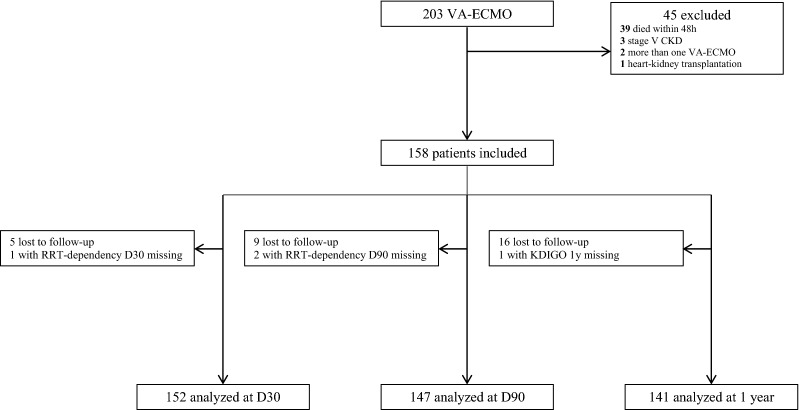
Flowchart

Between 2014 and 2016, 203 patients received VA-ECMO during their ICU stays. 39 patients (19%) died within 48 h after implementation and 6 other patients had an exclusion criterion. One hundred and fifty-eight patients were included (male sex: 76%; median and interquartile range: age: 59 [47–66], baseline serum creatinine 91 [71–106] (17% of missing data requiring an imputation), baseline eGFR 78 [62–101], Simplified Acute Physiology Score II: 55 [39–66], Sepsis-related Organ Failure Assessment Score: 9 [7–12], time on VA-ECMO: 7.5 [4–12] days). Among them 145 (91.8%) develop an AKI during the intensive care unit (ICU) stay and 85 (54%) needed renal replacement therapy (RRT). Finally 152, 147 and 141 patients were included for the D30, D90 and 1-year analysis, respectively. At 1 year, 17 patients (10.1%) patients were lost to follow-up at 1 year (none undergoing RRT at last news) (flowchart: Fig. 1). The characteristics of the patients according to the vital status at 1 year are in Table 1. The median duration of ECMO support was 7.5 [4; 12] days.

**Table 1:** Population characteristics

	All patients (*n* = 158)	Without MAKE-360 (*n* = 21)	With MAKE-360 (*n* = 120)
Women/men	24.1/75.9	2/19	33/87
Weight (kg)	77 [65; 90]	74 [65; 85]	78 [66; 90]
Height (cm)	170 [165; 178]	178 [169; 180.5]	170 [165; 177]
Body mass index (kg/m^2^)	26.2 [22.9; 30]	24.4 [20.6; 26.4]	26.8 [23.8; 30.5]
Age (years)	59 [47; 66]	49 [39; 56]	60 [49.5; 66.5]
Length of ICU stay (days)	20.5 [12; 39]	19 [14; 39]	21 [12; 38.5]
SAPS-II at ICU admission	54.5 [39; 66]	39 [21; 60]	55.5 [40.5; 68]
SOFA at ICU admission	9 [7; 12]	8 [5; 10]	9.5 [7; 12]
Respiratory item	1 [0; 2]	1 [0; 2]	2 [1; 3]
Neurologic item	0 [0; 1]	0 [0; 2]	0 [0; 1]
Hemodynamic item	4 [3; 4]	4 [2; 4]	4 [3; 4]
Liver item	1 [0; 2]	1 [0; 2]	1 [0; 2]
Kidney item	1 [0; 2]	0 [0; 1]	1 [0; 2]
Coagulating item	1 [0; 2]	0 [0; 2]	1 [0; 2]
Known comorbidities			
Previous myocardial infarction	45 (28.5)	2 (9.5)	37 (30.8)
Chronic cardiac failure	93 (58.9)	7 (33.3)	77 (64.2)
Peripheral vascular disease	19 (12)	1 (4.8)	16 (13.3)
Dementia	1 (0.6)	0 (0)	1 (0.8)
Cerebrovascular disease	20 (12.7)	1 (4.8)	18 (15)
Chronic respiratory disease	29 (18.4)	2 (9.5)	24 (20)
Connectivitis	6 (3.8)	3 (14.3)	2 (1.7)
Ulcers	11 (7)	0 (0)	10 (8.3)
Chronic liver disease	3 (1.9)	0 (0)	3 (2.5)
Diabetes	44 (27.8)	3 (14.3)	38 (31.7)
Hemiplegia	2 (1.3)	0 (0)	2 (1.7)
Moderate-to-severe renal failure	34 (21.5)	0 (0)	32 (26.7)
Solid tumor	6 (3.8)	0 (0)	6 (5)
Leukemia	1 (0.6)	1 (4.8)	0 (0)
Lymphoma	1 (0.6)	0 (0)	1 (0.8)
AIDS	0	0	0
Hypertension	62 (39.2)	3 (14.3)	53 (44.2)
Active smoking	23 (14.6)	4 (19)	17 (14.2)
Dyslipidemia	50 (31.6)	3 (14.3)	41 (34.2)
Alcoholism	10 (6.3)	3 (14.3)	6 (5)
Duration of CBP (min)*	148.5 [109; 210]	140 [89; 177]	155 [116; 220]
No CBP	90 (60)	11 (55)	71 (62.8)
CBP ≤ 140 min	28 (18.7)	4 (20)	20 (17.7)
CBP > 140 min	40 (25.3)	5 (25)	22 (19.5)
Duration of aortic clamping (min)*	81.5 [56.5; 114.5]	102.5 [55; 127]	81 [56; 111]
Place of cannulation			
In ICU	29 (18.4)	1 (4.8)	22 (18.3)
Operative bloc	116 (73.4)	18 (85.7)	88 (73.3)
Other	13 (8.2)	2 (9.5)	10 (8.3)
Cannulation site			
Femoro-femoral	66 (41.8)	10 (47.6)	45 (37.5)
Other	92 (58.2)	11 (52.4)	75 (62.5)
Time from ICU admission to cannulation (days)	1 [1; 1]	1 [1; 1]	1 [1; 2]
ECMO duration (days)	7.5 [4; 12]	6 [3; 8]	8 [5; 13]
Recannulation	31 (19.6)	3 (14.3)	28 (23.3)
Mechanical ventilation at cannulation	123 (77.8)	16 (76.2)	92 (76.7)
SOFA at cannulation	9 [7; 12]	9 [6; 10]	9 [7; 12]
Respiratory item	2 [1; 3]	2 [1; 2]	2 [1; 3]
Neurological item	0 [0; 4]	0 [0; 4]	0 [0; 4]
Hemodynamic item	4 [4; 4]	4 [4; 4]	4 [4; 4]
Liver item	0 [0; 2]	1 [0; 2]	0 [0; 2]
Kidney item	1 [0; 2]	0 [0; 1]	1 [0; 2]
Coagulation item	0 [0; 1]	0 [0; 1]	0 [0; 1]
SOFA without hemodynamic score	5 [3; 8]	5 [3; 6]	5 [3; 8]
Biological features at cannulation			
Lactate (mmol/l)	3 [2.2; 5.4]	3.1 [3; 6.1]	3 [2.2; 4.5]
AST (UI/l)	93.5 [47; 262]	94 [60; 437]	93.5 [45.5; 276.5]
ALT (UI/l)	68 [35; 216]	68 [36; 289]	68 [34.5; 186]
Platelets count (g/l)	163 [123; 228]	187 [104; 232]	162 [123; 225]
≤ 50 g/l	7 (4.4)	2 (9.5)	5 (4.2)
> 50 g/l	61 (38.6)	6 (28.6)	48 (40)
> 150 g/l	90 (57)	13 (61.9)	67 (55.8)
Hemoglobin (g/dl)	10 [7.9; 12.3]	9.8 [7.2; 10.4]	10 [8; 12.6]
≤ 10 g/dl	81 (51.3)	11 (52.4)	61 (50.8)
> 10 g/dl	77 (48.7)	10 (47.6)	59 (49.2)
Leukocytes (g/l)	10.9 [6.9; 16.4]	11.5 [8; 14.6]	10.5 [6.8; 16.3]
PT (%)	58 [41; 75]	60 [49; 74]	58 [40; 74]
aPTT ratio	1..4 [1.2; 1.7]	1.2 [1.1; 1.4]	1.4 [1.2; 1.8]
Hemorrhagic shock	79 (50)	8 (38.1)	64 (53.3)
Other than hemopericardium	43 (27.2)	5 (23.8)	35 (29.2)
Early hemopericardium	24 (15.2)	1 (4.8)	21 (17.5)
Late hemopericardium	12 (7.6)	2 (9.5)	8 (6.7)
Number of RBC	9 [5; 19]	5 [4; 10]	12 [6; 22]
ECMO duration (days)	7.5 [4; 12]	6 [3; 8]	8 [5; 13]
Outcomes			
Bridge to VAD	10 (6.3)	2 (9.5)	8 (6.7)
Bridge to heart transplantation	19 (12)	2 (9.5)	17 (14.2)
ECMO weaning	80 (50.6)	17 (81)	46 (38.3)
No weaning	49 (31)	0 (0)	49 (40.8)

Results are shown as median and interquartile ranges for quantitative variables and number and percentage for qualitative variables

*Duration of CBP and duration of aortic clampling apply only for patients who underwent CBP

*AIDS* acquired immuno deficiency syndrome, *CBP* cardiopulmonary bypass, *SAPS* simplified acute physiology score, *SOFA* sequential organ failure assessment, *ALT* alanine aminotransferase, *AST* aspartate aminotransferase, *aPTT* activated partial thromboplastin time, *ECMO* extracorporeal membrane oxygenation, *ICU* intensive care unit, *PT* prothrombin time, *RBC* red blood cell, *VAD* ventricle assistance device

**Fig. 2:**
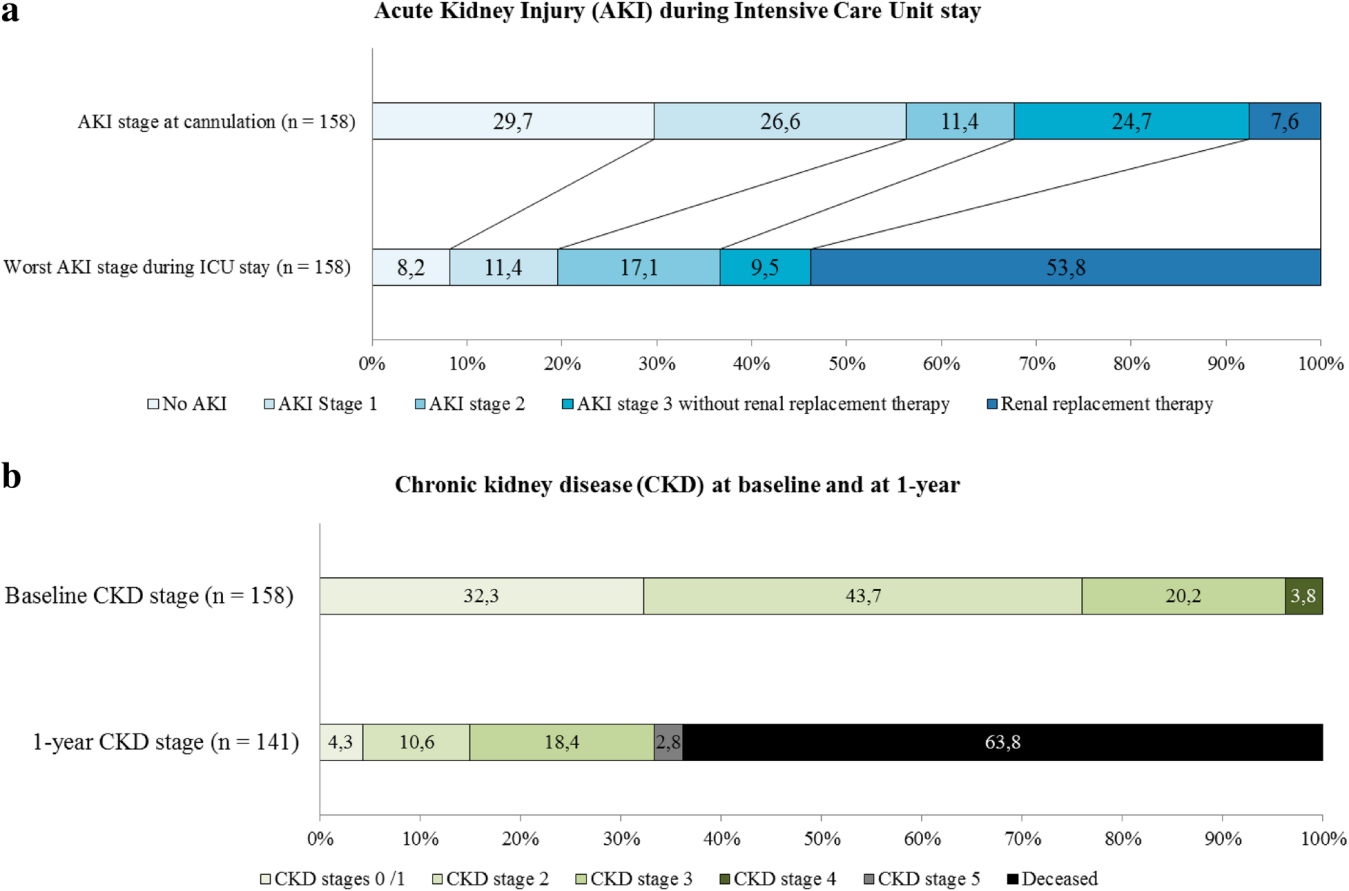
**a**, **b** Evolution of renal function during ICU stay and within a year

At baseline, 51 (32.3%) patients had no significant CKD (eGFR over 90 ml/min/1.73 m^2^). The median baseline eGFR was 78.3 [62.2–101] ml/min/1.73 m^2^. The repartition of CKD stages at baseline is described in Fig. 2.

### AKI prevalence

**Fig. 3:**
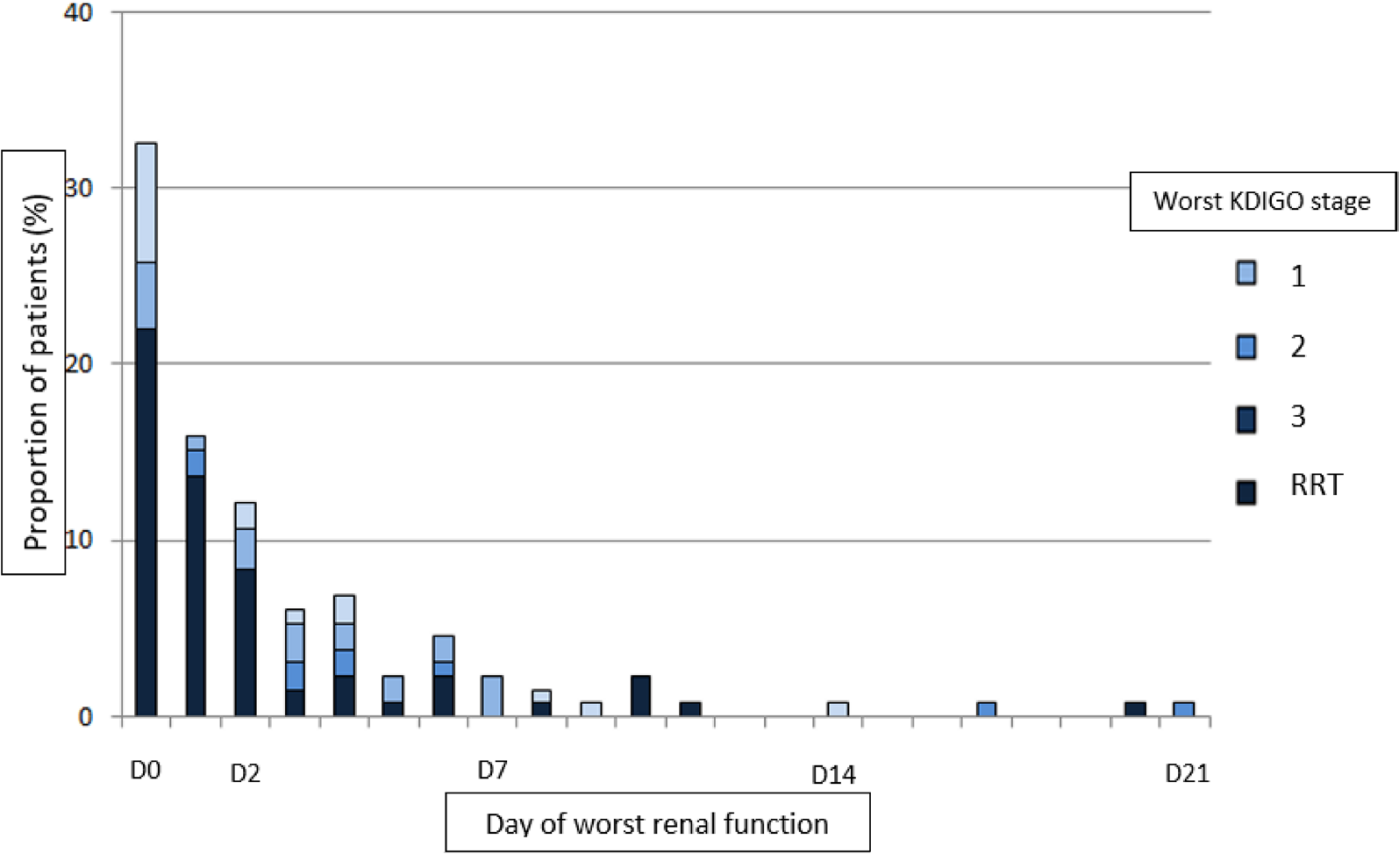
Day of worst renal function. *KDIGO* Kidney Disease Improving Global Outcome, *RRT* renal replacement therapy

Only 13 patients (8.2%) did not develop any AKI during ICU stay according to KDIGO classification and 85 (53.8%) patients needed RRT within ICU stay (Fig. 2a). The worst KDIGO occurred in the first days of VA-ECMO therapy for most patients 58 (95%) reached their worst KDIGO stage within 2 days after cannulation and 142 (89.9%) within the first week (Fig. 3).

### Factors associated with primary and secondary outcomes (Table 2)

Factors significantly associated with MAKE-360 were eGFR at baseline (odds ratio (OR) 0.98, confidence interval 95% (CI) [0.97;1.00], *p* 0.02), KDIGO stage at cannulation (*p* = 0.03), e.g., stage 3 vs. reference stage 0 OR 10.20 [1.77–58.87], and number of red blood cell (RBC) packs received while under ECMO (OR 1.14, CI 95% [1.01;1.28], *p* = 0.03). At 1 year among the 51 survivors, almost half of the alive patients (*n* = 20/51) had a decline of estimated glomerular filtration (eGFR) > 30% ml/min/1.73 m^2^. Their median eGFR decline was − 26.3% [− 46.6;− 10.7]. The only factor independently and constantly associated with MAKE whatever the evaluation timeline was the number of RBC packs received while under ECMO, OR 1.09 CI 95% [1.03–1.15], *p* < 0.01 at day 30 and OR 1.08 CI 95% [1.02–1.15], *p* < 0.01 at day 90.

**Table 2:** Multivariate logistic regression analysis: independent predictors of major adverse kidney events (MAKE) at day 30, at day 60, and at day 360

Variables	MAKE30^a^		MAKE90^a^		MAKE360^b^	
	OR [95% CI]	*p* value	OR [95% CI]	*p* value	OR [95% CI]	*p* value
eGFR at baseline (by mL/min/1.73 m^2^)	0.99 [0.98–1.00]	0.25	0.99 [0.98–1.00]	0.04	0.98 [0.97–1.00]	0.02
KDIGO stage at cannulation		< 0.01		0.12		0.03
Stage 0	1		1		1	
Stage 1	3.58 [1.28–10.00]		1.31 [0.47–3.60]		4.47 [0.99–20.20]	
Stage 2	4.95 [1.28–19.12]		3.26 [0.78–13.61]		3.04 [0.51–18.14]	
Stage 3	5.73 [2.12–15.50]		2.86 [1.07–7.65]		10.20 [1.77–58.87]	
SOFA score without hemodynamic and renal items	1.06 [0.91–1.23]	0.43	1.06 [0.91–1.23]	0.46	0.87 [0.69–1.09]	0.23
aPTT ratio at cannulation	1.05 [0.68–1.60]	0.83	1.38 [0.86–2.20]	0.17	1.17 [0.57–2.39]	0.67
Duration of CBP		0.38		0.08		0.69
Duration of CBP ≥ 140 min	1		1		1	
No CBP	1.98 [0.74–5.29]		3.01 [1.11–8.17]		1.89 [0.45–7.97]	
Duration of CBP < 140 min	1.68 [0.55–5.12]		1.51 [0.49–4.62]		1.44 [0.28–7.47]	
Number of RBC packs received while under ECMO	1.09 |1.03–1.15]	< 0.01	1.08 [1.02–1.15]	< 0.01	1.14 [1.01–1.28]	0.03
ECMO duration	0.99 [0.93–1.06]	0.78	1.03 [0.95–1.10]	0.48	1.04 [0.92–1.17]	0.54

Adjustment on eGFR at baseline, KDIGO stage at cannulation, SOFA score without hemodynamic and renal items, aPTT ratio at cannulation, duration of CBP, number of RBC packs while under ECMO, ECMO duration

*OR* odds ratio, *IC* interval confidence, *VA-ECMO* veno-arterial extracorporeal membrane oxygenation, *RRT* renal replacement therapy, *OR* odds ratio, *CI* confidence interval, *SOFA* sepsis-related organ failure assessment score, *KDIGO* kidney disease: improving global outcomes, *RBC* red blood cells, *aPTT* activated partial thromboplastin time, *CBP* cardiopulmonary bypass, *eGFR* estimated glomerular filtration rate

^a^Defined as one of the following criteria within day 30 or day 90: death, receipt of renal replacement therapy and serum creatinine ≥ threefold increase

^b^Defined as one of the following criteria within 1 year: death and receipt of renal replacement therapy (RRT) or persistent renal dysfunction, i.e., CKD ≥ stage 3 corresponding to an eGFR by MDRD ≤ 60 ml/min/1.73 m^2^

### Long-term follow-up

Ninety-two (60.5%), 70 (47.6%), and 51 (38.9%) of patients were alive at D30, D90 and at 1 year, respectively. Among patients who died in ICU, 31 (43%) were not weaned of RRT at the time of death. Only 4 (6.7%) of the alive patients at ICU discharge were on RRT after the 1-year follow-up, three of them did not require RRT at ICU discharge but experienced recurrent AKI during follow-up.

The repartition of CKD stages at 1 year is described in Fig. 2. Among the 51 survivors at 1 year about half of them (26/51 (51%)) had a CKD stage ≥ 3 and almost half of them (*n* = 20/51) had a decline of estimated glomerular filtration (eGFR) > 30% ml/min/1.73 m^2^. Their median eGFR decline was − 26.3% [− 46.6;− 10.7] corresponding to a median absolute decline of − 19.8 [− 50.8; − 4.8] ml/min/1.73 m^2^.

## Discussion

To date, only a few studies have evaluated the prevalence of AKI in patients undergoing VA-ECMO and long-term renal function of these patients was never reported. Among 158 patients included in our study, 145 (91.8%) develop an AKI during the intensive care unit (ICU) stay and 85 (53.8%) needed renal replacement therapy (RRT). 59.9% (91/152), 60.5% (89/147) and 85.1% (120/141) evaluable patients had a MAKE-30, MAKE-90 and MAKE-360, respectively. Factors significantly associated with MAKE-360 were eGFR, at baseline, KDIGO stage at cannulation and, number of RBC received while under ECMO. Our results documented also that among survivors of VA-ECMO therapy, long-term renal impairment is major with a median eGFR decline rate of 26.3% (− 20 ml/min/1.73 m^2^) at 1 year and half of those patients had a decline > 30%. Those results are of paramount importance since this technique is now commonly used worldwide.

AKI occurs very frequently in patients undergoing VA-ECMO. The prevalence in our study was about 92% while the prevalence of AKI in ICU patients has been estimated between 25 and 50% and between 70 and 85% in patients who underwent ECMO [12–16]. Comparing our findings with the latter results is challenging since the classification used, the population involved and the type of ECMO support were different.

We found that baseline renal function was associated with MAKE at 1 year. In a large cohort of patients older than 67 years with hospital-associated AKI who survived to discharge, Ischani et al. [17], found that the risk of chronic RRT was increased by nearly 7 times. Moreover, the risk was accentuated in patients with preexisting kidney disease. AKI seems to independently predispose a patient to long-term renal impairment. However, this assumption is based on data coming from administrative registries. Wald et al. [18] conducted a population-based cohort study of all adult patients in Ontario, Canada. They reported an incidence of ESRD of 2.63 and 0.91 cases per 100 ICU person-year in patients with AKI requiring dialysis during the ICU stay (*n* = 3769), and in matched controls without AKI (*n* = 13,589), respectively. Given the small number of patients who undergo VA-ECMO annually in each center and the lack of long-term follow-up for these patients, such an analysis is not suitable. Consequently, we used a surrogate marker, i.e., decline in eGFR to assess the risk of ESRD. This surrogate outcome has been described by Coresh et al. [19], who analyzed individual meta-analysis of 1.7 million participants from 35 cohorts of CKD and high cardiovascular risk population. They found that a decline in eGFR of 30% was associated with more than fivefold in the risk of ESRD. In our population, half of the patients had such a decline. To our knowledge, no study investigates eGFR decline in ICU patients, which does not allow us to compare our result. Nonetheless, the median decline rate over 1 year in our population of patients was striking. Use of MDRD-based eGFR is debatable in our critically ill patients with important changes in muscular mass during acute and recovery phases, but we would argue that in those, Scr would lower and eGFR might be overestimated which magnify the possible result in our cohort [20].

The magnitude of the eGFR decline allows understanding that any second renal hit can precipitate patients to ESRD. As a matter of fact, in our study, ESRD at 1 year was rare, but occurred in patients with recurrent AKI. This pattern, i.e., relapsing without recovery, was previously described in a study that examined the different patterns for AKI reversal in critically ill patients. It represents 14.7% of the 16,968 patients studied [21].

An unexpected finding of this study was that RBC packs transfusion was associated with MAKE whatever the evaluation timeline. Aubron et al. [14] found that mortality increased with the number of RBC packs transfused in patients who underwent ECMO while surgery for hemorrhage itself was not associated with an increased risk of death. It is not clear how RBC transfusion decreases survival or increase renal function impairment in such patients. One of the reasons could be hemolysis resulting from the combination of already altered red cell viability after storage with a technique well-known for inducing hemolysis by itself. Hemolysis occurs frequently in patients who undergo ECMO. It occurs in at least 5 to 10% of patients when measured using free hemoglobin according to ELSO report (*Extra*-*corporeal Life Support Organization*) [22] and depends on various technical aspects (size of cannulas, rotation speed). In a cohort study of 50 pediatric patients requiring ECMO, Borasino et al. recently found that hemolysis was associated with prolonged need of RRT and death after discharge [23]. Excessive hemolysis can oversaturate the hemoglobin clearance pathways resulting in cell-free hemoglobin and iron overload and related toxicity. There is a growing body of evidence that acute iron overload can cause serious adverse events such as clinically relevant AKI [24]. In adults, the association of RBC packs transfusion and AKI has been extensively described in cardiac surgery with CBP [25] and in other settings [26].

Results obtained in our study should be tempered by several limitations. First, our data reflect the experience of a single center potentially limited external validity. Second, we evaluated a mixed population of patients, who have received VA-ECMO following medical, postcardiotomy or post-cardiac arrest cardiogenic shock. A detailed evaluation of each specific population might be the focus on future studies. Third, although these analyses are based on the totality of our experience over 3 years, the total number of patients who underwent VA-ECMO is not high and our analyses could have been underpowered for some analyses. Fourth, many patients were lost to follow-up at 1 year and taking into consideration the high mortality rate of this population, these losses may have had a great impact on the results. Fifth, we did not collect data on anticoagulation and hemoglobin levels prior to transfusion, we did not monitor plasma free hemoglobin or other hemolysis parameters and due to retrospective data collection, we might lack information on potential unmeasured confounders.

## Conclusion

Patients receiving VA-ECMO are at risk to develop AKI within the ICU stay and had a severe eGFR decline at 1 year.
